# Integrating HDAd5/35++ Vectors as a New Platform for HSC Gene Therapy of Hemoglobinopathies

**DOI:** 10.1016/j.omtm.2018.02.004

**Published:** 2018-02-15

**Authors:** Chang Li, Nikoletta Psatha, Hongjie Wang, Manvendra Singh, Himanshu Bhusan Samal, Wenli Zhang, Anja Ehrhardt, Zsuzsanna Izsvák, Thalia Papayannopoulou, André Lieber

**Affiliations:** 1 Division of Medical Genetics, Department of Medicine, University of Washington, Box 357720, Seattle, WA 98195, USA; 2 Division of Hematology Department of Medicine, University of Washington, Seattle, WA 98195, USA; 3 Department of Pathology, University of Washington, Seattle, WA 98195, USA; 4 Max-Delbrück-Center for Molecular Medicine, Berlin, 13092 Germany; 5 Witten/Herdecke University, Witten, 58448, Germany

**Keywords:** hematopoietic stem cells, Sleeping Beauty transposase, beta-thalassemia, sickle cell disease, gamma globin, integration, gene addition

## Abstract

We generated an integrating, CD46-targeted, helper-dependent adenovirus HDAd5/35++ vector system for hematopoietic stem cell (HSC) gene therapy. The ∼12-kb transgene cassette included a β-globin locus control region (LCR)/promoter driven human γ-globin gene and an elongation factor alpha-1 (EF1α)-mgmt^P140K^ expression cassette, which allows for drug-controlled increase of γ-globin-expressing erythrocytes. We transduced bone marrow lineage-depleted cells from human CD46-transgenic mice and transplanted them into lethally irradiated recipients. The percentage of γ-globin-positive cells in peripheral blood erythrocytes in primary and secondary transplant recipients was stable and greater than 90%. The γ-globin level was 10%–20% of adult mouse globin. Transgene integration, mediated by a hyperactive *Sleeping Beauty* SB100x transposase, was random, without a preference for genes. A second set of studies was performed with peripheral blood CD34^+^ cells from mobilized donors. 10 weeks after transplantation of transduced cells, human cells were harvested from the bone marrow and differentiated *ex vivo* into erythroid cells. Erythroid cells expressed γ-globin at a level of 20% of adult α-globin. Our studies suggest that HDAd35++ vectors allow for efficient transduction of long-term repopulating HSCs and high-level, almost pancellular γ-globin expression in erythrocytes. Furthermore, our HDAd5/35++ vectors have a larger insert capacity and a safer integration pattern than currently used lentivirus vectors.

## Introduction

### HSC Gene Therapy

Hemoglobinopathies (β-thalassemia and sickle cell disease) are the most frequent monogenic diseases worldwide, with approximately 5% of the world population carrying a hemoglobin disorder trait.^1^ Current hematopoietic stem cell (HSC) gene therapy for hemoglobinopathies involve self-inactivating lentivirus (SIN-LV)-based vectors expressing β-globin (either wild-type or anti-sickling variants of β-globin) or the fetal form γ-globin. Therapeutic genes are under the control of β-globin locus control region (LCR) versions for erythroid-specific, position-independent expression. For HSC transduction, SIN-LV-globin vectors are incubated *ex vivo* with CD34^+^ cells for 2 to 3 days under conditions that support cell cycling. *Ex vivo* transduced HSCs are then transplanted into myelo-conditioned recipients. Trials for β-thalassemia and sickle cell disease showed a good safety profile and resulted in a significant reduction of transfusion requirements for beta^0^/beta^0^ thalassemia patients and improved quality of life.^2^, ^3^, ^4^, ^5^, ^6^ These studies also revealed a number of problems. Because the vectors require an erythroid-specific LCR, SIN-LV vectors for globin gene therapy are relatively large and therefore difficult to produce at high titers. This in turn influences the cost for gene therapy. Because of the structure and/or size of globin SIN-LV vectors, the HSC transduction frequency is relatively low.^5^ Although no leukemic events have been found in patients treated with SIN-LV vectors, their preference for integrating into active genes and transformation events seen *in vitro* after HSC transduction^7^ create a challenge for HSC gene therapy.

### HDAd5/35++ Vectors

According to a 2017 count of clinical gene therapy trials, adenovirus vectors are the most often used vectors in clinical gene therapy trials (21.2%) (http://abedia.com/wiley/vectors.php). In the context of our work on Ad biology, we identified CD46 as the high-affinity receptor for a number of Ads, including serotype 11, 16, 21, 35, and 50.^8^ CD46 is expressed on all human HSCs.^9^ Recently, we have shown that CD46 is expressed at higher levels on mouse and human HSCs than on more differentiated bone marrow and blood cells.^10^ The receptor-interacting moiety in the capsid of adenoviruses is the C-terminal globular trimeric fiber domain, called the fiber knob. We and others have shown that adenovirus vectors containing the Ad35 fiber or fiber knob (Ad5/35) efficiently transduce HSCs *in vitro*.^11^, ^12^, ^13^ Ad5/35 vector transduction of HSCs did not require cell division.^11^ The incoming Ad vector genome is bound to viral proteins that mediate nuclear import.^14^ To avoid HSC cytotoxicity associated with first-generation Ad5/35 vectors,^15^ we generated helper-dependent HDAd5/35++ vectors devoid of all viral genes. These vectors contained a series of mutations in the Ad35 fiber knob that increased the affinity to CD46 more than 25-fold and allowed for more efficient cell transduction at lower MOIs.^16^ The production of HDAd5/35++ vectors involves the co-transfection of a plasmid containing the HDAd vector genome and a helper virus that provides all structural and non-structural viral proteins but cannot be packaged. Virus rescue is done at a small scale (6-cm plates). The virus is produced at a large scale in a 2-l spinner culture that routinely yields >1 × 10^13^ viral particles (vp) per spinner.^17^ The purified virus preparation can then be used as a stock for further large-scale production. Based on our experience, HDAd5/35++ production can easily be established following the protocol by D. Palmer and P. Ng.^17^

HDAd5/35++ vectors do not integrate into the cellular genome. This, however, is a requirement for HSC gene therapy because an episomal vector would be lost after several cell divisions. We therefore incorporated a *Sleeping Beauty* transposase-based system that mediates transgene integration. This system consists of an HDAd5/35++ transposon vector that carries the transgene expression cassette, which is flanked by inverted transposon repeats (IRs) and flippase recognition target (FRT) sites. The second HDAd5/35++ vector provides both Flpe recombinase and an activity-enhanced *Sleeping Beauty* transposase (SB100x)^18^ *in trans*. Upon co-infection of both vectors, Flpe mediates the circularization of the transposon through the FRT sites. SB100x then randomly integrates the transposon into the host genome through interaction with the IRs.

In a recent study, we tested SB100x-armed HDAd5/35++ vectors for *in vivo* HSC transduction.^10^, ^19^ The approach involved the subcutaneous injection of granulocyte-colony stimulating factor (GCSF)/AMD3100 to mobilize HSCs from the bone marrow into the peripheral blood stream and the intravenous injection of the integrating HDAd5/35++ vector system. We demonstrated in adequate mouse models that our vectors allow for the stable transduction of HSCs, with a preference for transducing primitive HSCs (Lin^–^/Sca-1^+^/c-Kit^+^ [LSK] cells, colony-forming unit [CFU], long-term repopulating cells).^10^ 30 weeks after *in vivo* transduction, GFP marking in bone marrow HSCs was in the range of 5%–10%. The percentage of GFP-expressing primitive HSCs capable of forming multi-lineage progenitor colonies (CFUs) increased from 4% of all CFUs at week 4 to 16% at week 12, indicating transduction and expansion of long-term surviving HSCs. We found that the majority of GFP^+^ HSCs in the bone marrow are quiescent, not efficiently contributing to downstream differentiation. We used an *in vivo* HSC chemo-selection approach to give gene-modified HSCs a proliferation stimulus.^20^ This system is based on a mutant of the O^6^-methylguanine-DNA methyltransferase (mgmt^P140K^) gene that confers resistance to methylating agents (e.g., O^6^-benzylguanine [O^6^-BG] plus bis-chloroethylnitrosourea [BCNU] or temozolomide).^21^, ^22^, ^23^ We showed in mobilized, *in vivo* transduced mice that 4 cycles of O^6^-BG/BCNU treatment resulted in stable GFP expression in ∼80% of peripheral blood cells.^20^

Here, we generated an integrated HDAd5/35++ vector with a 11.8-kb transgene cassette containing a 5-kb β-globin LCR/promoter version controlling the expression of a full-length γ-globin gene as well as an elongation factor alpha-1 (EF1α)-promoter driven mgmt^P140K^ expression cassette. We studied γ-globin expression after mouse and human HSC *ex vivo* transduction and subsequent transplantation into myeloablated recipients. These studies are an important step toward our final goal, which is *in vivo* HSC gene therapy of hemoglobinopathies.

## Results

### HDAd-γ-Globin/mgtm Vector

**Figure 1:**
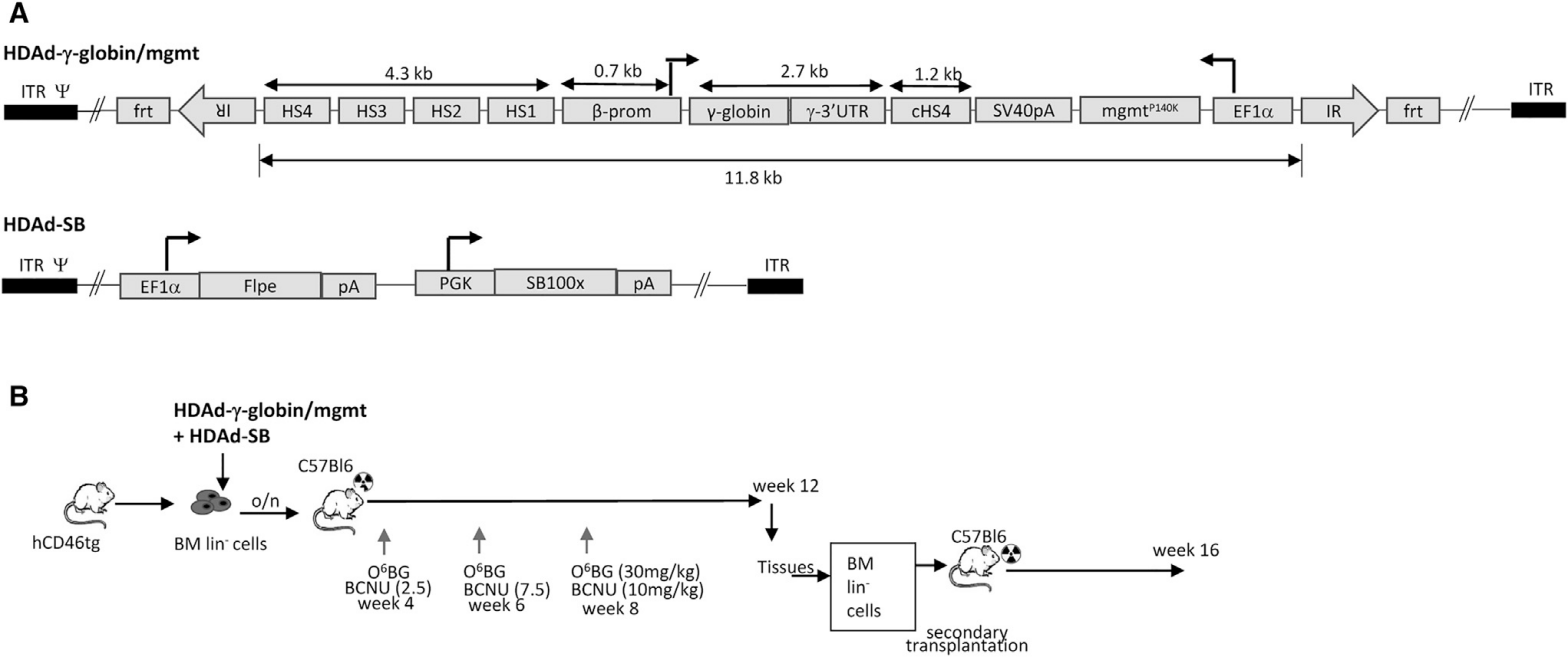
Integrating HDAd5/35++ Vector (A) Vector structure. In HDAd-γ-globin/mgtm, the 11.8-kb transposon is flanked by inverted transposon repeats (IRs) and FRT sites for integration through a hyperactive *Sleeping Beauty* transposase (SB100x) provided from an HDAd-SB vector.^10^ The γ-globin expression cassette contains a 4.3-kb version of the β-globin LCR consisting of four DNase hypersensitivity (HS) regions and the β-globin promoter.^51^ The full-length γ-globin cDNA, including that 3′ UTR (for mRNA stabilization in erythrocytes) was used. The mgmt^P140K^ gene is under the control of the ubiquitously active EF1α promoter. The bidirectional SV40 poly-adenylation signal is used to terminate transcription. To avoid interference between the LCR/β-promoter and EF1α promoter, a 1.2-kb chicken HS4 chromatin insulator^25^ was inserted between the cassettes. The second vector (HDAd-SB) provides both Flpe recombinase and the SB100x transposase *in trans*. Both vectors are helper-dependent adenovirus (HDAd) vectors containing the CD46 affinity-enhanced Ad35++ fiber knob and Ad35 fiber shaft.^16^ Upon co-infection of both vectors, Flpe mediates the circularization of the transposon through FRT sites. SB100x then randomly integrates the transposon into the host genome through interaction with the IRs. (B) Schematic of the experiment. Bone marrow was harvested from hCD46tg mice and lineage-negative cells (Lin^–^) were isolated by magnetic-activated cell sorting (MACS). Lin^−^ cells were transduced with HDAd vectors at a total MOI of 1,000 vp/cell at a ratio of 1:1. After 1 day in culture, 1 × 10^6^ transduced cells/mouse were transplanted into lethally irradiated C57BL/6 mice. At week 4, O^6^-BG/BCNU treatment was started and repeated every 2 weeks for a total of 3 times. With each cycle, 30 mg/kg O^6^-BG was applied, whereas the BCNU dose was increased from 5 mg/kg to 7.5 mg/kg and 10 mg/kg. Animals were sacrificed at week 12, and bone marrow Lin^−^ cells were transplanted into secondary recipients, which were then followed for 16 weeks.

For β-thalassemia gene therapy to be curative, it is essential that the transferred γ-globin gene be expressed in erythroid cells at high levels, without position effect of integration and transcriptional silencing. To achieve this, the β-globin LCR is required.^24^ Here, we used a 5.0-kb β-globin LCR version that contained the key DNase I hypersensitivity (HS) HS1–HS4 regions and the β-globin promoter (Figure 1A, upper panel). The LCR is driving a full-length human γ-globin (HBG1) gene, including the 3′ UTR, which stabilizes γ-globin RNA in (enucleated) erythrocytes. There are two known variants of the HBG1 gene in the human population with a single amino acid variation (76-Isoleucine or 76-Threonine). We used in our studies the 76-Ile HBG1 variant, which has a range in frequency of 13% in Europeans to 73% in East Asians. The vector also contained an mgmt^P140K^ expression cassette mechanism for *in vivo* selection. The mutant mgmt gene is under the control of the ubiquitously active EF1α promoter, therefore allowing for selection at the level of HSCs, progenitors, and differentiated cells. The *in vivo* selection system will make it possible to maximize the frequency of γ-globin-expressing erythrocytes. A 1.2-kb chicken HS4 chromatin insulator^25^ was inserted between the γ-globin and mgmt^P140K^ expression cassettes to avoid promoter interference. In the HDAd5/35++ γ-globin/mgmt vector, the transposon to be integrated into the host genome will have a length of 11.8 kb. The HDAd-SB vector that provides the hyperactive SB100x transposase *in trans* (Figure 1A, lower panel) has been described recently.^10^ In this study, the HDAd-γ-globin/mgmt and HDAd-SB vectors were produced at a titer of ∼1 × 10^13^vp/mL, without detectable genomic rearrangements.

### Studies in hCD46 Transgenic Mice

HDAd5/35++ vectors infect cells through CD46. Although CD46 is expressed on all nucleated cells in humans, the corresponding ortholog in mice is expressed only in the testes. As a model for our *in vivo* HSC transduction studies, we used a C57BL/6-based transgenic mouse model (hCD46tg) that contained the complete human CD46 locus. These mice express hCD46 in a pattern and at a level similar to those of humans.^26^ We used hCD46tg mice as a source for HSCs in this study (Figure 1B). For *ex vivo* transduction, bone marrow cells depleted for differentiated progenitor cells that were positive for lineage markers (CD5, CD45R, CD11b, Gr-1, 7-4, and Ter119) were used. Lineage-negative (Lin^−^) cells are enriched for HSCs. Immediately after isolation, Lin^−^ cells were transduced with HDAd-γ-globin/mgmt + HDAd-SB and incubated overnight in medium that did not support cell proliferation (“low cytokine” medium). The *ex vivo* incubation was kept as short as possible to preserve the multipotency of HSCs. We used an MOI of 1,000 vp/cell, an MOI that resulted in efficient Lin^−^ cell transduction in previous studies with HDAd5/35++ vectors expressing GFP.^10^, ^20^ A total of 5 × 10^5^ transduced Lin^−^ cells were transplanted into lethally irradiated C57BL/6 mice. C57BL/6 mice do not express human CD46, which allows us to follow engraftment of transplanted cells based on hCD46 by flow cytometry. 4 weeks after transplantation, mice received three cycles of O^6^-BG/BCNU treatment and were sacrificed at week 12 (“primary recipients”). Tissues were analyzed for γ-globin expression. To demonstrate the stable genetic modification of long-term repopulating HSCs, Lin^−^ cells from primary recipients were again transplanted and secondary recipients were followed for 16 weeks (Figure 1B).

**Figure 2:**
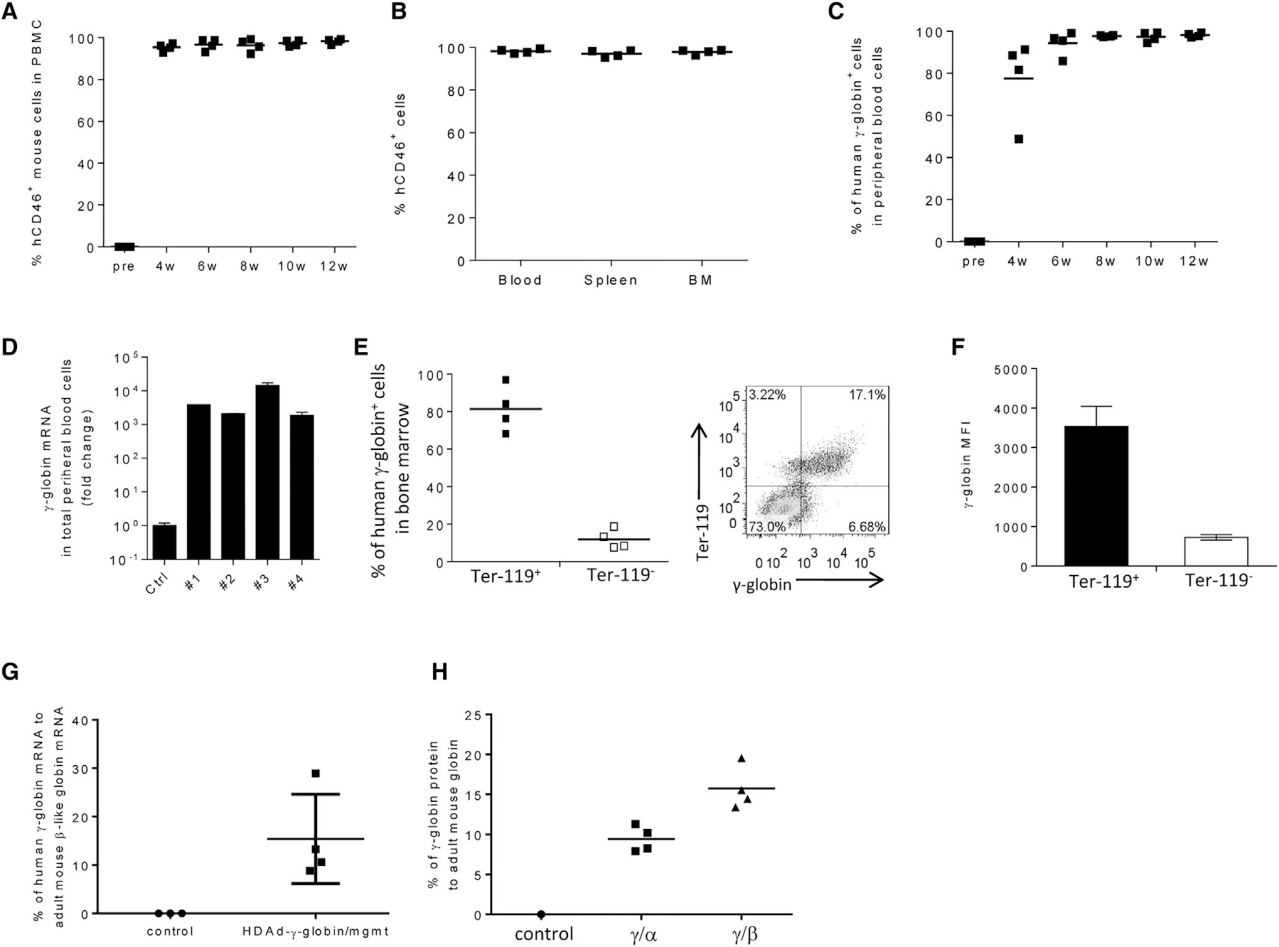
Analysis of Primary Transplanted Mice (A) Engraftment of transplanted cells based on human CD46 expression on PBMCs measured by flow cytometry. Each symbol is an individual animal. (B) Engraftment rates in blood, spleen, and bone marrow mononuclear cells analyzed at week 12 after transplantation. (C) Percentage of human γ-globin-positive cells in peripheral blood cells measured by flow cytometry. (D) Fold increase in γ-globin mRNA in total peripheral blood cells in four treated mice (#1–#4) compared to β-YAC mice. (E–H) Analysis of week 12 bone marrow samples. (E) γ-globin expression in erythroid (Ter-119^+^) and non-erythroid (Ter-119^−^) cells. The right panel shows a representative sample. (F) Mean fluorescence intensity (MFI) of γ-globin in Ter-119^+^ and Ter-119^−^ cells. (G) Percentage of human γ-globin mRNA relative to mouse adult β-like globin RNA. Controls are bone marrow samples from untreated C57BL/6 mice. (H) HPLC data. Percentage of human γ-globin protein relative to mouse adult α- and β-globin protein.

Analysis of blood cells collected at weeks 4, 6, 8, 10, and 12 from primary recipients showed over 99% engraftment of transplanted cells (Figure 2A). This was confirmed in spleen and bone marrow samples analyzed at week 12 after transplantation (Figure 2B). Expression of γ-globin was analyzed by flow cytometry through intracellular staining with an antibody specific for human γ-globin. At 4 weeks, on average, ∼80% of blood cells expressed γ-globin (Figure 2C). Notably, 99.8% of blood cells are erythrocytes. Therefore, the frequency of γ-globin-positive total blood cells is representative for erythrocytes. The latter was confirmed by flow cytometry for γ-globin and the erythroid cell marker Ter-119. After initiation of *in vivo* selection, the percentage of γ-globin-expressing blood erythrocytes reached nearly 100% and was stable over the observation period of 12 weeks. γ-globin mRNA levels were three to four orders of magnitude higher in HDAd-γ-globin/mgmt erythrocytes compared to untreated β-YAC mice^27^ (Figure 2D). γ-globin expression was also analyzed in bone marrow cells that were positive or negative for the erythroid surface marker Ter-119 (Figures 2E and 2F). The percentage of γ-globin-expressing cells was ∼8-fold lower on Ter-119^−^ cells compared to Ter-119^+^ cells (Figure 2E). Lower expression levels in non-erythroid cells were also reflected in the mean fluorescence intensity (MFI) for γ-globin (Figure 2F). γ-globin expression levels in bone marrow cells were ∼15% of those of adult mouse (α- or β-like) globin at the level of mRNA (measure by qRT-PCR) (Figure 2G) and protein (measured by HPLC) (Figure 2H).

**Figure 3:**
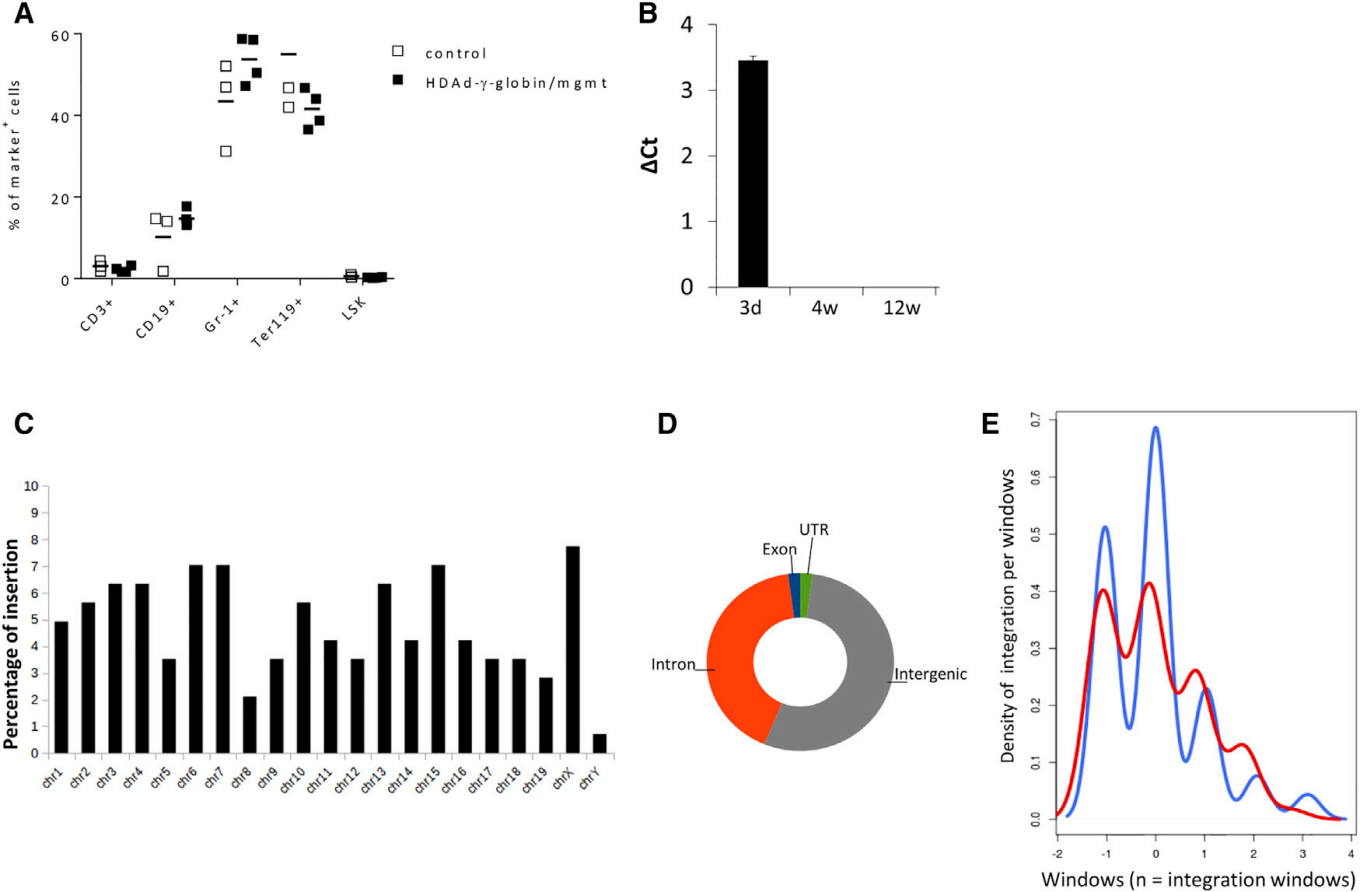
Safety Studies (A) Cellular bone marrow composition in naive mice (control) and treated mice sacrificed at week 12. Shown is the percentage of lineage marker positive cells (Ter-119^+^, CD3^+^, CD19^+^, and Gr-1^+^ cells) and HSCs (LSK cells). (B) SB100x mRNA levels in total bone marrow mononuclear cells measured by qRT-PCR in comparison to mRNA levels of the house-keeping gene mRPL10. ΔCt = Ct^SB100x^−Ct^mRPL10^. Samples were collected at day 3 after *in vitro* transduction of Lin^−^ cells and at weeks 4 and 12 after transplantation of HDAd-SB+HDAd-γ-globin/mgmt transduced Lin^−^ cells. No SB100x mRNA signals were detected at weeks 4 and 12. N = 3. (C) Chromosomal distribution of integration sites. Genomic DNA was isolated from total bone marrow mononuclear cells (week 12 samples pooled from 4 male mice). Shown is the distribution of integrations over the mouse genome displayed as a fraction of total integrations per chromosome. (D) Percentage of integrations in exons, untranslated regions (UTRs), and intronic and intergenic regions. (E) Pattern of integration in continuous (blue) and random mouse genomic windows (red). The similarity is statistically significant. Pearson’s Chi-square test: p = 0.1669.

To evaluate the potential myelotoxicity of the *in vivo* selection process, we studied the cellular composition in bone marrow at week 12 (Figure 3A). Analysis of the frequency of lineage cells showed no significant differences between untreated mice and mice that received transduced Lin^−^ cells and subsequent *in vivo* selection. The level of Lin^−^/Sca1^+^/cKit^+^ (LSK) HSCs was also comparable in both groups (∼0.5%).

To assess potential side effects of SB100x expression, we measured SB100x mRNA by qRT-PCR (Figure 3B). Although SB100x mRNA was clearly detectable in Lin^−^ cells in culture at day 3 after transduction, no detectable signals were found in bone marrow mononuclear cells at weeks 4 and 12 after transplantation. This indicates that the episomal HDAd-SB vector and, consequently, SB100x expression is rapidly lost during cell division in transplanted animals.

To analyze genotoxic effects due to SB100x-mediated transgene integration and subsequent *in vivo* selection, we performed a genome-wide integration analysis using linear amplification-mediated PCR (LAM-PCR), followed by Illumina sequencing of genomic DNA isolated at week 12 from bone marrow cells of primary recipients (Figures 3C–3E). SB100x-mediated transgene integration sites were randomly distributed over the mouse genome (Figure 3E). Integrations within exons constituted 2.1% of all integration events. 41.6% of integration sites were within introns, 54.2% in intergenic regions, and 2.1% in untranslated regions (Figure 3D). The pattern of integration is statistically similar in both continuous and random mouse genomic windows (Figure 3E).

In conclusion, SB100x-mediated random transgene integration showed no preference for genes. The *in vivo* selection process did not change the random integration pattern compared to previous studies without *in vivo* selection.^10^

**Figure 4:**
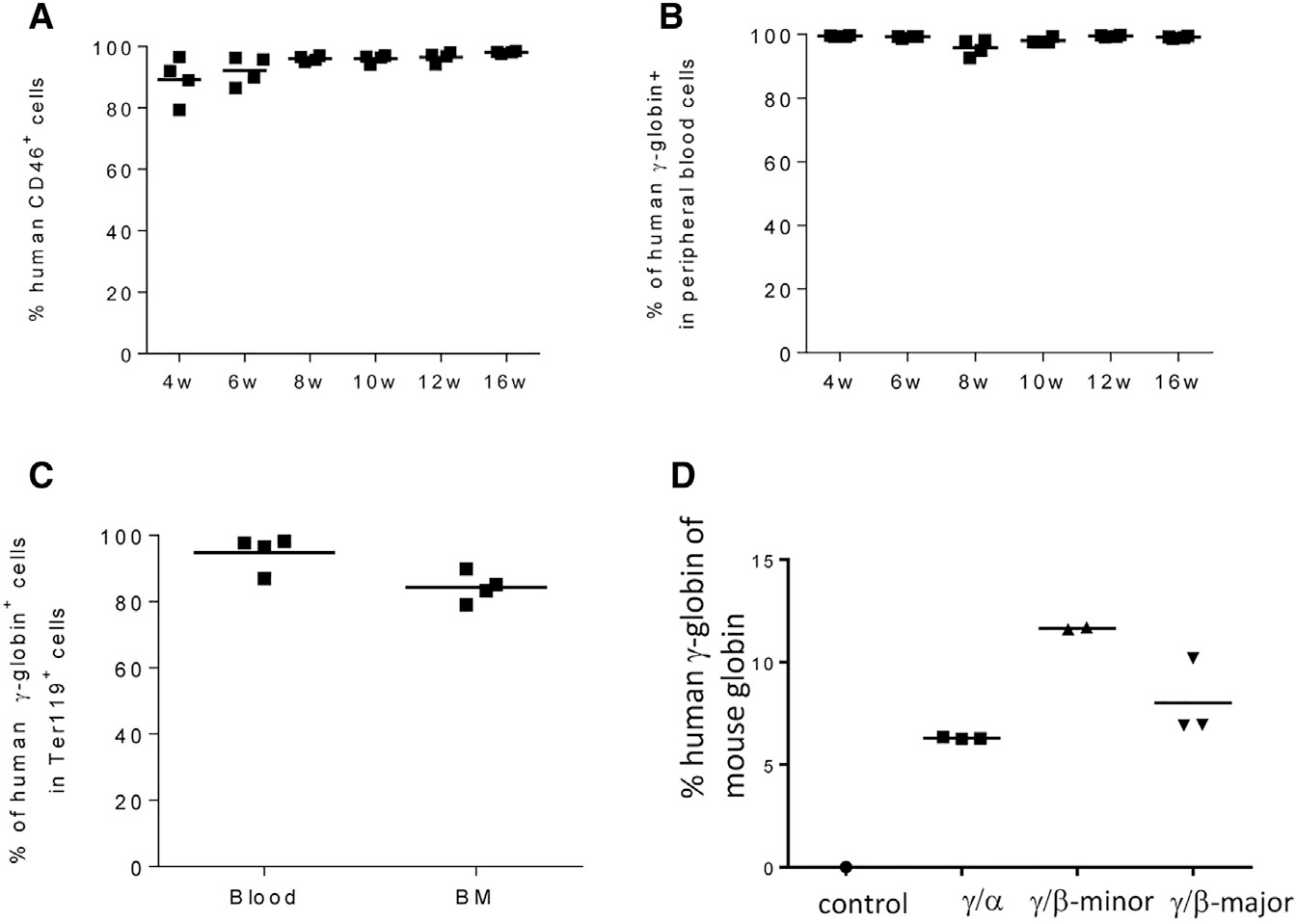
Analysis of Secondary Recipients (A) Engraftment based on hCD46 expression on PBMCs. (B) Percentage of human γ-globin-positive cells in peripheral blood cells. (C) Percentage of human γ-globin-positive cells in blood and bone marrow Ter119^+^ cells. (D) Percentage of human γ-globin protein relative to mouse adult α- and β-globin protein in total peripheral blood cells at week 16.

To further confirm that our approach resulted in genetic modification of primitive HSCs, we performed a transplantation/repopulation study in secondary recipients. Lineage-negative bone marrow cells from primary recipients collected at week 12 after transplantation were used for secondary transplantation into lethally irradiated C57BL/6 mice. Analysis of hCD46 expression on PBMCs at weeks 4–16 after secondary transplantation showed engraftment rates of >90% in all recipients (Figure 4A). The percentage of human γ-globin-positive peripheral red blood cells was also nearly 100%, indicating that genetically modified HSCs had repopulated the blood system (Figure 4B). The frequency of γ-globin-positive Ter-119^+^ cells in blood and bone marrow was in the range of 90%–95% (Figure 4C). The γ-globin expression level in blood cells, measured by HPLC, was in the range of 7%–12% of mouse adult globin (Figure 4D).

In summary, these data suggest that our vector system targets long-term repopulating cells and that these cells are maintained through the selection process.

### Studies with Human CD34^+^ Cells from G-CSF Mobilized Donors

**Figure 5:**
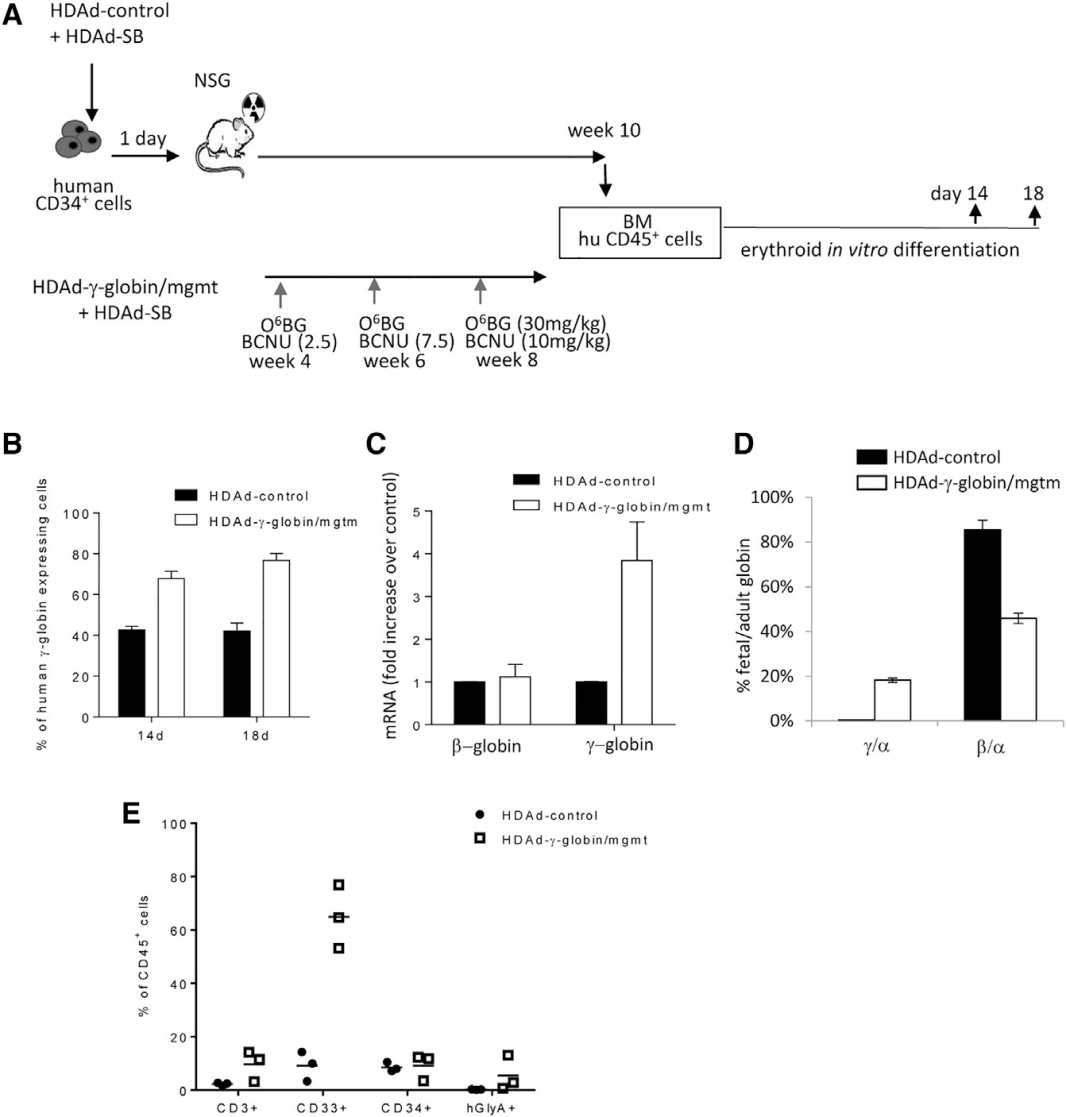
Studies with Human CD34+ Cells (A) Schematic of the experiment. Human CD34^+^ cells were transduced *ex vivo*, and 24 hr later, transplanted into sublethally irradiated NOG mice. Mice that received the HDAd-γ-globin/mgmt vector were treated with O^6^-BG/BCNU as indicated. 10 weeks after transplantation, mice were sacrificed, and bone-marrow-derived human CD34^+^ cells were subjected to erythroid *in vitro* differentiation. (B) Percentage of human cells expressing γ-globin at day 14 and 18 of erythroid differentiation. (C) Adult (β-globin) and γ-globin mRNA in cells at day 14 of *in vitro* differentiation. (D) HPLC for globin chains. γ/α-globin and beta/α-globin percentages at day 18 of erythroid differentiation. (E) Percentage of human-lineage-marker-positive cells in the bone marrow at week 9 after transplantation.

To increase the relevance of our studies with integrating HDAd5/35++ vectors for HSC gene therapy, we performed studies with human CD34^+^ cells, a cell fraction that is enriched for HSCs (Figure 5A). 16 hr after recovery of CD34+ cells from cryopreserved stocks in low-cytokine medium, cells were transduced with HDAd-γ-globin/mgmt+HDAd-SB at a total MOI of 2,000 vp/cell. This MOI conferred efficient CD34^+^ cell transduction in previous studies with HDAd5/35++ vectors.^10^, ^28^ To assess the effect of HDAd5/35++ transduction on activation of endogenous γ-globin expression in CD34^+^ cells, we also transduced cells with an HDAd5/35++ vector containing the human FVIII gene (HDAd-control). After overnight incubation with the vectors, CD34^+^ cells were transplanted into sublethally irradiated immunodeficient NSG mice. Engraftment rates measured at week 4 after transplantation, based on the hCD45 expression on PBMCs, were, on average, 10% for both groups. This is in the range that we observed with this CD34^+^ cell donor after transplantation of untransduced cells^15^, ^28^, ^29^ and suggests that HDAd5/35++ transduction does not reduce the engraftment potential of CD34^+^ cells. At week 4, 6, and 8 after transplantation, mice transplanted with HDAd-γ-globin/mgtm+HDAd-SB transduced HSCs received O^6^-BG/BCNU treatment. All animals were sacrificed at week 10. NSG mice do not support the differentiation of human HSCs into erythrocytes, which is required to achieve expression of the ectopic γ-globin gene from the β-globin LCR. We therefore isolated human CD45^+^ cells from the bone marrow of transplanted mice and incubated them in a medium that triggers their erythroid differentiation. In agreement with published data,^30^ a relatively high frequency of cells expressing endogenous γ-globin (40%) was observed after erythroid differentiation in HDAd-control transduced samples. A similar percentage of γ-globin-positive cells was observed at day 18 of erythroid differentiation of naive (untransduced) CD34^+^ cells recovered from cryopreservation (data not shown). This indicates that these culture conditions activate fetal globin synthesis in CD34^+^ cells. Importantly, the frequency of γ-globin-positive cells was significantly higher (75%–80%) in erythroid cells obtained from mice that were transplanted with HDAd-γ-globin/mgmt transduced HSCs (p < 0.01) (Figure 5B). γ-globin RNA levels were ∼4-fold higher in HDAd-γ-globin/mgmt samples than in HDAd-control samples, whereas mRNA levels for β-globin were comparable (Figure 5C). We also analyze levels of γ- and adult α- and β-globin chains by HPLC. The γ-to-α ratio in HDAd-γ-globin/mgtm samples suggests ectopic γ-globin expression at a level of 20% of α chains (Figure 5D). Interestingly, ectopic γ-globin expression decreased the levels of β-globin, as indicated by the ratio of β-to-α, most likely to keep the mean corpuscular hemoglobin concentration in the physiological range. Analysis of lineage-positive cells in the bone marrow at week 10 showed comparable frequencies in CD34^+^ cells but higher levels of CD33^+^, CD3^+^, and hGlyA^+^ (erythroid) cell fractions in the HDAd-γ-globin/mgmt group (Figure 5E), which was most likely the result of *in vivo* selection.

Overall, our data demonstrate efficient γ-globin expression after *ex vivo* HSC transduction with integrating HDAd5/35++ vectors and subsequent transplantation and erythroid differentiation.

## Discussion

Currently, SIN lentivirus and rAAV6 vectors are used for HSC transduction.^31^, ^32^, ^33^ Integrating HDAd5/35++ vectors have a number of potential advantages over SIN-LV and rAAV vectors for globin gene therapy. (1) The production of HD-Ad5/35++ vectors does not require large-scale plasmid transfection and yields high viral titers, which will greatly reduce the costs of clinical gene therapy. Based on our experience, starting from an HDAd5/35++ virus stock and using the manufacturing protocol developed by Philip Ng,^17^ the costs for laboratory-grade HDAd5/35++ vector is about $600-$1,000 per 1 × 10^13^ viral genomes. For comparison, the production of the same amount of a rAAV vector would cost ∼10-fold more. Strimvelis, a lentivirus-based HSC gene therapy is sold for $600,000–$700,000 per patient. This price includes the complete current Good Manufacturing Practices (cGMP) procedure of HSC harvesting, transduction, and transplantation. (2) In contrast to lentivirus vectors, HDAd5/35++ vectors do not require cell cycling for transduction.^11^ (3) Although the maximum insert capacity of SIN-LV and rAAV vectors is ∼8 and 5 kb, respectively, HDAD5/35++ vectors have an insert capacity of 30 kb. This is relevant for reaching curative levels of therapeutic globin, which is thought to require the full-length (26 kb) β-globin LCR.^24^ (4) Integration is mediated by the SB100x transposase system, which functions independently of cellular factors. (5) HDAd5/35++ vectors armed with the SB100x-based integration system do not have a preference of transgene integration into active genes.^10^ In contrast, both lentivirus and rAAV vectors have a preference for integrating into active genes.^33^, ^34^

Here, we demonstrated the utility of integrating HDAd5/35++ vector for gene therapy of hemoglobinopathies. We generated an integrated HDAd5/35++ vector with an 11.8-kb transgene cassette containing a 5-kb β-globin LCR/promoter version controlling the expression of a full-length γ-globin gene as well as an EF1α-mgmt^P140K^ expression cassette. The 11.8-kb transgene cassette would not fit into SIN-LV vectors, which have an insert capacity of ∼8.5 kb. We incorporated the mgmt^P140K^-based *in vivo* selection system into our vector because this would allow for drug-controlled increase of the γ-globin level if levels start declining and patients become transfusion-dependent again, a problem seen with globin-expressing SIN-LV vectors. Focusing on introducing the γ-globin gene rather than the β-globin gene has the advantage that it can be used for gene therapy of both β-thalassemia and sickle cell disease.

In a first set of studies, we transduced bone marrow Lin^−^ cells from hCD46tg *ex vivo* and transplanted them into lethally irradiated recipients. Engraftment rates were ∼100% and stable, indicating that vector transduction did not negatively affect the viability and function of HSCs. The expression of γ-globin in primary and secondary recipients in peripheral blood erythrocytes and erythroid cells in the bone marrow was almost pancellular (90%–100%). The γ-globin level was 10%–20% of adult mouse globin. In previous studies with γ-globin lentivirus vectors, these levels resulted in an amelioration of murine β-thalassemia and sickle cell diseases under conditions in which γ-globin-expressing erythroid cells have a survival advantage.^35^, ^36^, ^37^ Stable γ-globin expression in primary and secondary recipients and the demonstration of random transgene integration by genome-wide sequencing suggest (1) that the HDAd535++ vector system targeted long-term repopulating HSCs with high efficiency. This is potentially due to the facts that the vector uses CD46 for infection, a receptor that is expressed at higher levels on primitive HSCs than on more differentiated bone marrow cells and that it can transduce non-dividing HSCs. (2) The SB100x can integrate an 11.8-kb transposon. So far, SB100x-mediated integration had only been shown for transposons smaller than 6 kb. (3) The integrated γ-globin transgene cassette escapes gene silencing. (4) The integration site analysis indicates that the *in vivo* selection process did not change the random integration pattern, i.e., did not result in clonal dominance.

Our studies showed γ-globin background expression in non-erythroid cells. This can potentially be addressed by replacing the 5-kb LCR with the 26-kb version. Potentially, the 26-kb LCR version can mediate higher γ-globin levels, which is of importance for the treatment of sickle cell disease. Notably, we have generated HDAd5/35 vectors containing the 26-kb LCR before.^38^ However, these vectors were not armed with the SB100x-based integration system. Further improvements could also include the modification of the HDAd5/35++ vectors for targeted integration into a single pre-selected site.^39^

Because mice received lethal irradiation and thus developed tolerance to foreign transgene products, vector/transgene immunogenicity has to be considered in settings with only partial myeloablation. In this context, it has to be noted that HDAd5/35++ vectors do not express viral proteins and that the SB100x-encoding vector is lost after several cell divisions. Also, the use of human transgenes (γ-globin and mgmt) should not trigger corresponding immune responses in patients.

A second set of studies was performed with peripheral blood CD34^+^ cells from mobilized healthy donors. Transduced cells were transplanted into sublethally irradiated NSG mice. Because this model does not support the differentiation of human HSCs into differentiated erythroid cells, engrafted human HSCs were harvested from the bone marrow at week 10 after transplantation and incubated *ex vivo* with cytokines that support erythroid differentiation. At the end of the differentiation process, we detected ectopic γ-globin expression at a level that was 20% of that of adult α-globin. In a previous *in vitro* study with γ-globin-expressing SIN-LV vectors, these levels were considered to be therapeutic in erythroid progeny of β-thalassemia CD34^+^ cells.^40^

There is no clinical experience with HDAd5/35++ vectors, and studies in non-human primates are still ongoing (A.L., unpublished data). First-generation Ad5/35 vector has been used in clinical trials for *ex vivo* T cell transduction.^41^, ^42^ Helper-dependent vectors derived from serotype 5 (HDAd5) have been tested in mice, dogs, and non-human primates for the treatment of diseases, including Apoplipoprotein A (ApoE) deficiency, atherosclerosis, α1 anti-trypsin deficiency, cystic fibrosis, Duchenne muscular dystrophy, glioblastoma, and infectious diseases (for a review, see Rosewell et al.^43^ and Brunetti-Pierri et al.^44^). There is a published report on the clinical use of HDAd5 vectors.^45^ Furthermore, Aevi Genomic Medicine’s website indicates clinical development of a HDAd vector expressing the metabotronic glutamate receptor for treatment of ADHD.

In summary, although our focus is still on developing our *in vivo* HSC gene therapy approach,^10^, ^19^, ^20^ the studies presented here suggest that our vectors are valuable for *ex vivo* HSC gene therapy of hemoglobinopathies. Setting up the clinical grade manufacturing of HDAd5/35++ vectors is a major focus of our upcoming efforts.

## Materials and Methods

### Generation of Transposon Vector HDAd-γ-Globin/mgmt

The shuttle plasmid pBS-μLCR was generated by DNA synthesis (GenScript, Piscataway, NJ). This plasmid consists of the core region of four erythroid-specific DNase I hypersensitive sites, HS4 (http://genome.ucsc.edu/), human chromosome 11: 5,288,038→5,289,100), HS3 (chromosome 11 [Chr11]: 5,284,251→5,285,551), HS2 (Chr11: 5,280,523→5,281,381), and HS1 (5,275,644→5,276,717), the human β-globin promoter (Chr11:5,227,022→5,227,684), and the chromatin insulator cHS4. Human γ-globin gene and its 3′ UTR region (Chr11:5,247,139→5,249,804)^46^ (a generous gift from Dr. Qiliang Li) was then inserted into pBS-μLCR downstream of the human β-promoter. The human EF1α promoter-mgmt(p140k)-SV40pA cassette was inserted downstream of the cHS4 (pBS-μLCR-γ-globin-mgmt). The expression cassette μLCR-γ-globin-mgmt was then released by PacI and inserted into plasmid pHM5-T/Ef1α-GFP-FRT2^10^ by replacing the EF1α-GFP cassette (pHM5-T/μLCR-γ-globin-mgmt-FRT2). The transposon contained the γ-globin and mgmt. genes. Expression cassettes flanked by FRT sites were then released by I-CeuI/PI-SceI restriction enzyme digestion and inserted into a modified pWE15-HCA plasmid (pWEHCA-μLCR-γ-globin-mgmt-FRT2). The pWE-HCA plasmid^15^ was modified by inserting I-CeuI/PI-SceI sites into the ClaI site. The resulting plasmids were packaged into phages using Gigapack III plus packaging Extract (Agilent Genomics) and propagated. To generate HDAd-γ-globin/mgmt virus, the viral genome were released by FseI digestion from the plasmid for rescue in 116 cells^47^ with AdNG163-5/35++, an Ad5/35++ helper vector containing chimeric fibers composed of the Ad5 fiber tail, the Ad35 fiber shaft, and the affinity-enhanced Ad35++ fiber knob.^10^

The generation of HDAd-SB has been described previously.^10^ Helper virus contamination levels were below 0.05%. All preparations were free of bacterial endotoxin.

### CD34^+^ Cell Culture

CD34^+^ cells from G-CSF-mobilized adult donors were recovered from frozen stocks and incubated overnight in Iscove’s modified Dulbecco’s medium (IMDM) supplemented with 10% heat-inactivated fetal calf serum (FCS), 1% BSA, 0.1 mmol/L 2-mercaptoethanol, 4 mmol/L glutamine and penicillin/streptomycin, Flt3 ligand (Flt3L, 25 ng/mL), interleukin-3 (IL-3) (10 ng/mL), thrombopoietin (TPO) (2 ng/mL), and stem cell factor (SCF) (25 ng/mL). Cytokines and growth factors were from Peprotech (Rocky Hill, NJ). CD34^+^ cells were transduced with virus in low attachment 12-well plates.

### Lin^−^ Cell Culture

Lineage-negative cells were isolated from total mouse bone marrow cells by MACS using the Lineage Cell Depletion kit from Miltenyi Biotech (Bergisch Gladbach, Germany). Lin^−^ cells were cultured in IMDM supplemented with 10% FCS, 10% BSA, Pen-Strep, glutamine, 10 ng/mL human TPO, 20 ng/mL mouse SCF, and 20 ng/mL human Flt3L.

### Erythroid *In Vitro* Differentiation

Differentiation of human HSCs into erythroid cells was done based on the protocol developed by Douay et al.^48^ In brief, in step 1, cells at a density of 10^4^ cells/mL were incubated for 7 days in IMDM supplemented with 5% human plasma, 2 IU/mL heparin, 10 μg/mL insulin, 330 μg/mL transferrin, 1 μM hydrocortisone, 100 ng/mL SCF, 5 ng/mL IL-3, 3 U/mL erythropoietin (Epo), glutamine, and Pen-Strep. In step 2, cells at a density of 1 × 10^5^ cells/mL were incubated for 3 days in IMDM supplemented with 5% human plasma, 2 IU/mL heparin, 10 μg/mL insulin, 330 μg/mL transferrin, 100 ng/mL SCF, 3 U/mL Epo, glutamine, and Pen/Strep. In step 3, cells at a density of 1 × 10^6^ cells/mL were incubated for 12 days in IMDM supplemented with 5% human plasma, 2 IU/mL heparin, 10 μg/mL insulin, 330 μg/mL transferrin, 3 U/mL Epo, glutamine, and Pen-Strep.

### Globin HPLC

Individual globin chain levels were quantified on a Shimadzu Prominence instrument with an SPD-10AV diode array detector and an LC-10AT binary pump (Shimadzu, Kyoto, Japan). A 40%–60% gradient mixture of 0.1% trifluoroacetic acid in water/acetonitrile was applied at a rate of 1 mL/min using a Vydac C4 reversed-phase column (Hichrom, UK).

### Flow Cytometry

Cells were resuspended at 1 × 10^6^ cells/100 μL in PBS supplemented with 1% FCS and incubated with FcR blocking reagent (Miltenyi Biotech, Auburn CA) for 10 min on ice. Next, the staining antibody solution was added in 100 μL per 10^6^ cells and incubated on ice for 30 min in the dark. After incubation, cells were washed once in FACS buffer (PBS, 1% FBS). For secondary staining, the staining step was repeated with a secondary staining solution. After the wash, cells were resuspended in FACS buffer and analyzed using a LSRII flow cytometer (BD Biosciences, San Jose, CA). Debris was excluded using a forward scatter area and sideward scatter-area gate. Single cells were then gated using a forward scatter height and forward scatter-width gate. Flow cytometry data were then analyzed using FlowJo (version 10.0.8). For flow analysis of LSK cells, cells were stained with biotin-conjugated lineage detection cocktail (Miltenyi Biotec, San Diego, CA) and antibodies against c-Kit and Sca-1 as well as allophycocyanin (APC)-conjugated streptavidin. Other antibodies from eBioscience (San Diego, CA) included anti-mouse LY-6A/E (Sca-1)-PE-Cyanine7 (clone D7), anti-mouse CD117 (c-Kit)-PE (clone 2B8), anti-mouse CD3-APC (clone 17A2), anti-mouse CD19-PE-Cyanine7 (clone eBio1D3), and anti-mouse Ly-66 (Gr-1)-PE (clone RB6-8C5). Other antibodies from Miltenyi Biotec included anti-human CD45-APC (clone 5B1), anti-human CD3-PE/Cy7 (clone REA613), anti-human CD34-PE (clone AC136), and anti-human Glycophorin A-VioBlue (clone REA175). Anti-human CD19-PE (clone: HIB19) and anti-human CD33-BV421 (WM53) were from BD Biosciences. Anti-mouse Ter-119-APC (clone: Ter-119) was from BioLegend (San Diego, CA).

### Intracellular Flow Cytometry Detecting Human γ-Globin Expression

The FIX and PERM cell permeabilization kit (Thermo Fisher Scientific) was used, and the manufacturer’s protocol was followed. Briefly, ∼1 × 10^6^ cells were resuspended in 100 μL FACS buffer (PBS supplemented with 1% FCS), 100 μL reagent A (fixation medium) was added and incubated for 2 to 3 min at room temperature, and 1 mL pre-cooled absolute methanol was then added, mixed, and incubated on ice in the dark for 10 min. The samples were then washed with FACS buffer and resuspended in 100 μL reagent B (permeabilization medium) and 1 μg hemoglobin γ antibody (Santa Cruz Biotechnology, cat# sc-21756 PE), incubated for 30 min at room temperature. After the wash, cells were resuspended in FACS buffer and analyzed. For erythroid and γ-globin double staining, cells were stained with APC anti-mouse Ter-119 antibody first, and then washed and proceeded with fixation medium as described above.

### Real-Time RT-PCR

Total RNA was extracted from 50 to 100 μL blood by using TrIzol reagent (Thermo Fisher Scientific) following the manufacturer’s phenol-chloroform extraction method. Quantitect reverse transcription kit (QIAGEN) and power SYBR green PCR master mix (Thermo Fisher Scientific) were used. Real-time qPCR was performed on a Steponeplus real-time PCR system (AB Applied Biosystems). The following primer pairs were used: mouse RPL10 (house-keeping) forward, 5′-TGAAGACATGGTTGCTGAGAAG-3′, and reverse, 5′-GAACGATTTGGTAGGGTATAGGAG-3′; human γ-globin forward, 5′-GTGGAAGATGCTGGAGGAGAAA-3′, and reverse, 5′-TGCCATGTGCCTTGACTTTG-3′; mouse β-major globin forward, 5′- ATGCCAAAGTGAAGGCCCAT-3′, and reverse, 5′- CCCAGCACAATCACGATCAT-3′, and human β-globin forward, 5′-CTCATGGCAAGAAAGTGCTCG-3′, and reverse, 5′-AATTCTTTGCCAAAGTGATGGG-3′. For qRT-PCR of SB100x mRNA, the following primers were used: SB100x forward, 5′-AGCCACTGCTCCAAAACCGACA-3′, and reverse, 5′-AAGCCTCCCCCTTCTTCCTCCA-3′.

### Integration Site Analysis

Amplification of genomic DNA junctions was performed by linear amplification-mediated PCR, and bioinformatic analysis of integration sites was performed as described previously.^10^

To analyze the sequencing data, sample-specific barcoded sequencing reads were demultiplexed using CASAVA, an Illumina software package. The quality of sequencing runs of resulting fastq files was evaluated using FastQC (http://www.bioinformatics.babraham.ac.uk/projects/fastqc). Reads starting with the barcode 5′-GTATGTAAACTTCCGACTTCAACTG-3′ that follows the TA dinucleotide, which is characteristic of SB integration, were aligned against the latest version of mouse reference genome (GRCm38/mm10 [December 2011]) using Bowtie2.^49^ Only reads that mapped exactly to a unique position in the reference genome were kept for further analysis. To analyze the distribution integrations, annotations of exons, and coding DNA sequence (CDS) of the corresponding reference, genome were downloaded and the percentage of integration sites overlapping with the given genomic coordinates was analyzed using BEDTools.^50^ We have indexed and created 142 normal, shuffled, and randomized windows of mouse genome and counted the number of integrations for each window and plotted the density.

### Animals

All experiments involving animals were conducted in accordance with the institutional guidelines set forth by the University of Washington. The University of Washington is an Association for the Assessment and Accreditation of Laboratory Animal Care International (AALAC)-accredited research institution, and all live animal work conducted at this university is in accordance with the Office of Laboratory Animal Welfare (OLAW) Public Health Assurance (PHS) policy, USDA Animal Welfare Act and Regulations, the Guide for the Care and Use of Laboratory Animals, and the University of Washington’s Institutional Animal Care and Use Committee (IACUC) policies. The studies were approved by the University of Washington IACUC (Protocol No. 3108-01).

The immunodeficient NOD/Shi-scid/IL-2Rγnull (NSG) mice were obtained from Jackson Laboratory (Bar Harbor, ME). For HSC transplantation, NSG recipient mice received 300 Rad whole-body irradiation. 2.5 × 10^5^ whole bone marrow cells of non-irradiated NOG mice were mixed with 6 × 10^5^ transduced human CD34^+^ cells and injected intravenously into recipient mice at 4 hr post irradiation. For transplantation into C57BL/6 mice, animals were irradiated with 1,000 Rad. 4 hr after irradiation, transduced HSCs were injected intravenously through the tail vein at 1 × 10^6^ cells per mouse.

### Statistical Analyses

For comparisons of multiple groups, one-way and two-way ANOVA with Bonferroni post-testing for multiple comparisons was employed. Statistical analysis was performed using GraphPad Prism version 6.01 (GraphPad Software, La Jolla, CA).

## Author Contributions

A.L. provided the conceptual framework for the study. C.L., N.P., H.W., Z.I., and A.L. designed the experiments. C.L., N.P., H.W., M.S., H.B.S., and W.Z. performed the experiments. A.E. provided critical material. T.P. provided comments. A.L. wrote the manuscript.

## Conflicts of Interest

The authors declare no competing financial interests.
